# Determinants of diagnostic yield and scan quality of [^18^F]FDG PET/CT in critically ill patients suspected of infection or inflammation of unknown origin

**DOI:** 10.1186/s13054-026-06005-w

**Published:** 2026-04-10

**Authors:** Jelle L. G. Haitsma Mulier, Bram van Leer, Joanne J. Beijer-Verduin, Harm-Jan S. de Grooth, Jules Lavalaye, Peter G. Noordzij, Nena Pater, Stefan E. Pool, Thijs C. D. Rettig, Cornelis P. van Stee, Janesh Pillay, Hans Balink, Olaf L. Cremer, Arthur J. A. T. Braat, Lennie P. G. Derde

**Affiliations:** 1 Department of Intensive Care Medicine, University Medical Center Utrecht, Utrecht, The Netherlands; 2 Julius Center for Health Sciences and Primary Care, University Medical Center Utrecht, Utrecht, The Netherlands; 3 Department of Critical Care, University Medical Centre Groningen, University of Groningen, Groningen, The Netherlands; 4 Department of Nuclear Medicine and Molecular Imaging, University Medical Centre Groningen, University of Groningen, Groningen, The Netherlands; 5 Department of Nuclear Medicine, St Antonius Hospital, Nieuwegein, The Netherlands; 6 Department of Anaesthesiology & Intensive Care, St Antonius Hospital, Nieuwegein, The Netherlands; 7 Department of Nuclear Medicine, Amphia hospital, Breda, The Netherlands; 8 Department of Anesthesiology & Intensive Care Amphia hospital, Breda, The Netherlands; 9 Department of Radiology and Nuclear Medicine, University Medical Center Utrecht, Utrecht, The Netherlands; 10 Department of Nuclear Medicine, Netherlands Cancer Institute, Amsterdam, The Netherlands

**Keywords:** [^18^F]FDG PET/CT, Infection of unknown origin, Preparation, Diagnostic yield, ICU

## Abstract

**Background:**

[^18^F]-fluorodeoxyglucose Positron Emission Tomography combined with Computed Tomography ([^18^F]FDG PET/CT) is widely used to detect focal inflammation. However, its diagnostic performance and utility in the ICU remain unclear. We aimed to identify factors influencing diagnostic yield and scan quality of [^18^F]FDG PET/CT in critically ill patients suspected of infection or inflammation.

**Methods:**

We retrospectively evaluated [^18^F]FDG PET/CT scans performed in adult ICU patients across four Dutch hospitals (2013–2023). The primary endpoint was diagnostic yield: new conclusive diagnoses and/or therapy changes within 72 h. Secondary endpoints were scan quality and adequacy of myocardial suppression. Logistic regression was used to identify determinants of each endpoint.

**Results:**

We analyzed 169 scans for 162 clinical indications in 151 patients. A conclusive diagnosis was established for 109 indications (67%), including 79 new diagnoses (49%), most frequently involving the musculoskeletal system (*n* = 39; 24%) and the respiratory tract (*n* = 31; 19%). [^18^F]FDG PET/CT prompted a therapy change in 70 indications (43%). Diabetes (aOR 0.35 [95% CI: 0.15–0.80]) and partial-body imaging versus total-body imaging (aOR 0.48 [CI 0.24–0.99]) were associated with fewer new diagnoses. A longer pre-scan ICU stay predicted therapy change (aOR 1.03 [1.01–1.05]). Higher BMI (aOR 1.08 [1.00–1.17]) and lower serum creatinine (log-transformed aOR 0.36 [0.20–0.68]) were associated with improved scan quality. Male sex (aOR 0.45 [0.21–0.96]) and pre-scan unfractionated heparin (aOR 0.43 [0.19–0.98]) were associated with suboptimal myocardial suppression.

**Conclusions:**

[^18^F]FDG PET/CT has a high diagnostic yield in critically ill patients. Diagnostic yield, scan quality and myocardial suppression are influenced by patient characteristics and preparation. Optimizing these factors may further enhance its utility in the ICU.

**Supplementary Information:**

The online version contains supplementary material available at 10.1186/s13054-026-06005-w.

## Introduction

Infections and inflammatory processes affect over half of critically ill patients [1] and substantially contribute to morbidity, mortality, hospitalization, and costs [2]. Accurate and timely identification of the infectious or inflammatory focus is essential for guiding targeted therapy, minimizing unnecessary antibiotic use, and improving clinical outcomes [3]. However, in some patients, the focus of persistent or recurrent inflammation remains unlocalized after extensive diagnostic work-up [4].

In non-critically ill patients, [^18^F]-fluorodeoxyglucose Positron Emission Tomography ([^18^F]FDG PET) combined with Computed Tomography (CT) has emerged as a valuable imaging modality for infection when conventional diagnostics have been inconclusive [5–8]. Its role in the ICU, however, is far less established, and its use remains limited [9]. Only a few studies, with small sample sizes, have explored its diagnostic yield in the ICU [4, 10–13]. Moreover, it is unknown which parameters are associated with diagnostic yield.

To reduce the background uptake of FDG and improve scan interpretability, patient preparation is essential when performing [^18^F]FDG PET/CT in the ICU [14]. While international guidelines recommend specific preparatory protocols to optimize scan performance, including fasting, dietary carbohydrate restriction, and controlled insulin administration [15], it remains challenging to optimize scan quality, especially in the ICU. Critically ill patients often experience hyperglycemia as a result of elevated inflammatory cytokines, physiological stress, use of corticosteroids, tube feeding, or parenteral nutrition [16]. In addition, they often receive intravenous insulin as part of standard care [17, 18]. Both elevated blood glucose and insulin administration alter FDG biodistribution in skeletal and cardiac muscle, resulting in lower image quality and consequently lower sensitivity [19, 20]. Additionally, because cardiac assessment on [^18^F]FDG PET/CT is often required to identify a source of infection or inflammation, adequate myocardial suppression is essential. This typically requires extended fasting periods and a low-carbohydrate diet, although the optimal fasting duration remains unknown [14, 21]. Moreover, the impact of patient preparation on scan quality and myocardial suppression has not been evaluated in critically ill patients.

This study aims to quantify the diagnostic yield of [^18^F]FDG PET/CT in ICU patients suspected of infection or inflammation of unknown origin (IUO) and to explore factors influencing diagnostic yield, scan quality, and myocardial suppression.

## Methods

### Study design, setting, and eligibility criteria

We conducted a retrospective multicenter cohort study in four Dutch ICUs – two academic and two non-academic teaching hospitals. Data were collected between December 2013 and 2023 at one academic hospital and between December 2017 and 2023 at the other hospitals. We included all [^18^F]FDG PET/CT scans performed in adult ICU patients that aimed to identify a focus of infection or inflammation. Scans were excluded if they were aborted for technical reasons or performed for indications unrelated to IUO (e.g., screening for malignancy).

### Primary outcome

Diagnostic yield of [^18^F]FDG PET/CT was defined as (1) the identification of a new diagnosis and/or (2) a therapy change within 72 h after the scan. A new diagnosis was defined as the identification of any infectious or inflammatory focus that either had not previously been suspected by the treating physician, or one that had been considered but could not be confirmed by conventional diagnostic modalities within the 7 days preceding the scan request. A therapy change was defined as the initiation, prolongation, or cessation of medication, a therapeutic intervention, or restrictions on care, occurring within 72 h after [^18^F]FDG PET/CT. Diagnostic yield was determined independently by four clinicians (JHM, BvL, NP, and CvS), through review of the PET/CT scan report and the electronic health record. In cases of ambiguity, discrepancies were resolved through consultation with a senior clinician (LD).

### Secondary outcomes

Scan quality was independently assessed by two experienced nuclear medicine physicians (AB and HB, with 14 and 20 years of experience), who were blinded to clinical data and scan preparation. Each scan was rated on a three-point ordinal scale (“poor”, “mediocre,” or “good”) based on signal-to-noise ratio, motion and attenuation artefacts, and FDG biodistribution. Scan quality was classified as *optimal* if both assessors rated the scan as “good,” and *suboptimal* if either assessor rated the scan as “mediocre” or “poor.” We did not include an additional “poor” category, as only six scans were rated as “poor/poor,” which was insufficient to construct a stable three-class model. Myocardial FDG suppression was scored as *optimal* only when both assessors judged suppression to be adequate; in all other cases, suppression was classified as *suboptimal*.

### Statistical analysis

Statistical analyses were primarily descriptive. Continuous variables were summarized using medians with interquartile ranges. Categorical variables were expressed as frequencies and percentages.

For the primary outcome, the number and proportion of scans resulting in a new diagnosis or therapy change were reported. Scan quality and myocardial suppression were reported as absolute and relative frequencies. In the absence of a reference standard for diagnosing the underlying cause of IUO, we did not calculate test characteristics such as sensitivity, specificity, or predictive values.

We used univariable and multivariable logistic regression to assess associations between patient- or preparation-related factors and the binary outcomes of new diagnosis, therapy change, scan quality, and myocardial suppression. Variable selection was informed by prior literature and clinical plausibility. For the analysis of diagnostic yield, we selected variables that were clinically relevant and available at the time of scan request. For the analysis of scan quality and myocardial suppression, we selected variables known or hypothesized to influence FDG-biodistribution or myocardial uptake. We deliberately avoided parameter selection procedures in multivariable analysis, since our aim was not to develop a parsimonious prediction model but rather to evaluate the potential effect of each prespecified factor on the outcomes.

Odds ratios (OR) and 95% confidence intervals (95% CI) were reported for all associations. Model assumptions were assessed for all logistic regression models, including linearity of the logit [22], multicollinearity [23], and residual diagnostics [24]. All analyses were performed using R version 4.5.0 [25].

## Results

**Fig. 1:**
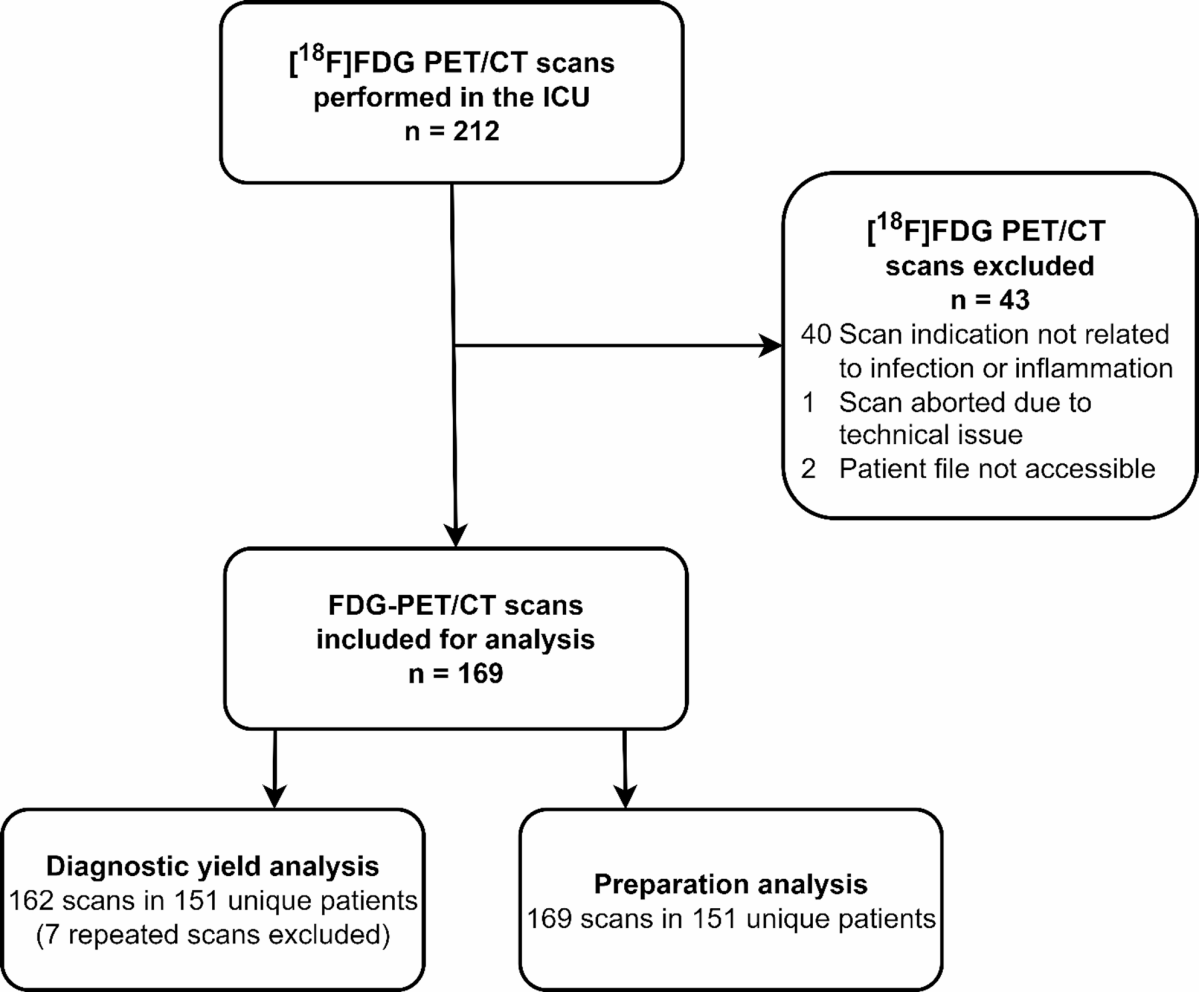
Flowchart of inclusion

During the study period, 212 [^18^F]FDG PET/CT scans had been performed in the participating ICUs, of which 169 met the eligibility criteria (Fig. 1). Eligible [^18^F]FDG PET/CT scans were performed for 162 indications among 151 unique patients. Seven scans were repeated for the same indication due to insufficient quality. Baseline characteristics are presented in Table 1. Complete data were available for all variables of interest. Approximately half of the patients (52%) underwent a total-body [^18^F]FDG PET/CT scan (vertex to toes) and 48% underwent a partial scan, most commonly extending from the skull to the groin. [^18^F]FDG PET/CT was performed after a median of 10 (IQR 5–22) days in the ICU. The most common indication was to identify the source of a suspected infection (134 indications, 83%). Eighteen indications (11%) concerned possible dissemination of a known infection, and 6 (4%) systemic inflammation without overt signs of infection. In a small number of patients, the scan was performed for follow-up of a previously diagnosed infection (3 patients, 2%) or in the context of unexplained respiratory and hemodynamic deterioration (1 patient, < 1%). The clinical status in the week preceding the scan request was indicative of ongoing inflammation in most patients (Table 1). The majority had elevated inflammatory markers, fever, and positive blood cultures, and nearly all patients received antibiotic therapy at the time of the [^18^F]FDG PET/CT scan.

**Table 1:** Characteristics of the included cohort

**1-A. Patient characteristics**	**n = 151**
Age	64 [50–71]
Male sex (at birth)	106 (70)
BMI	27.3 [24.3–30.3]
Comorbidities	
Diabetes mellitus	40 (26)
Immune deficiency	23 (15)
Any malignancy ^1^	25 (17)
Chronic renal insufficiency	17 (11)
Total length of ICU stay (days)	25 [11–50]
ICU mortality	44 (29)

**1-B. Clinical status at scan request (per indication)**	**n = 162**
CRP (mg/L)	172 [92.5–248]
White blood cell count (x10^9^/L)	14.3 [9.5–17.8]
Fever > 38.3 °C	106 (65)
Positive blood cultures	107 (66)
Antibiotic therapy	143 (88)
Duration of antibiotic therapy prior to scan	7 [4–13]
Type of nutritional intake Tube feeding Oral intake Other (e.g., total parenteral nutrition)	123 (76) 27 (17) 12 (7)
SOFA score	7 [4–9]

**1-C. Scan and preparation characteristics**	**n = 169**
Organ support on day of [^18^F]FDG PET/CT	
Mechanical ventilation	108 (64)
Vasopressors Renal Replacement Therapy	95 (56) 46 (27)
Preparation parameters Use of any corticosteroids in week before scan Any insulin administration on day of scan Pre-scan bolus of unfractionated heparin Pre-scan creatinine (µmol/L) ^2^ Pre-scan blood glucose (mmol/L) ^2^ Hours fasted before scan	78 (46) 44 (26) 49 (29) 93 [60–180] 6.3 [5.2–8.2] 24 [13–25]
Scan properties Total body scan [^18^F]FDG activity (MBq)	93 (55) 209 [157-265.5]
Time from request to scan (days)	2 [1–3]
ICU length of stay prior to [^18^F]FDG PET/CT	10 [5–22]

Presented as Median [IQR] or n (%)

^1^ Haematological or solid organ malignancy

^2^ Last measurement before scan

### Diagnostic work-up before [^18^F]FDG PET/CT

**Fig. 2:**
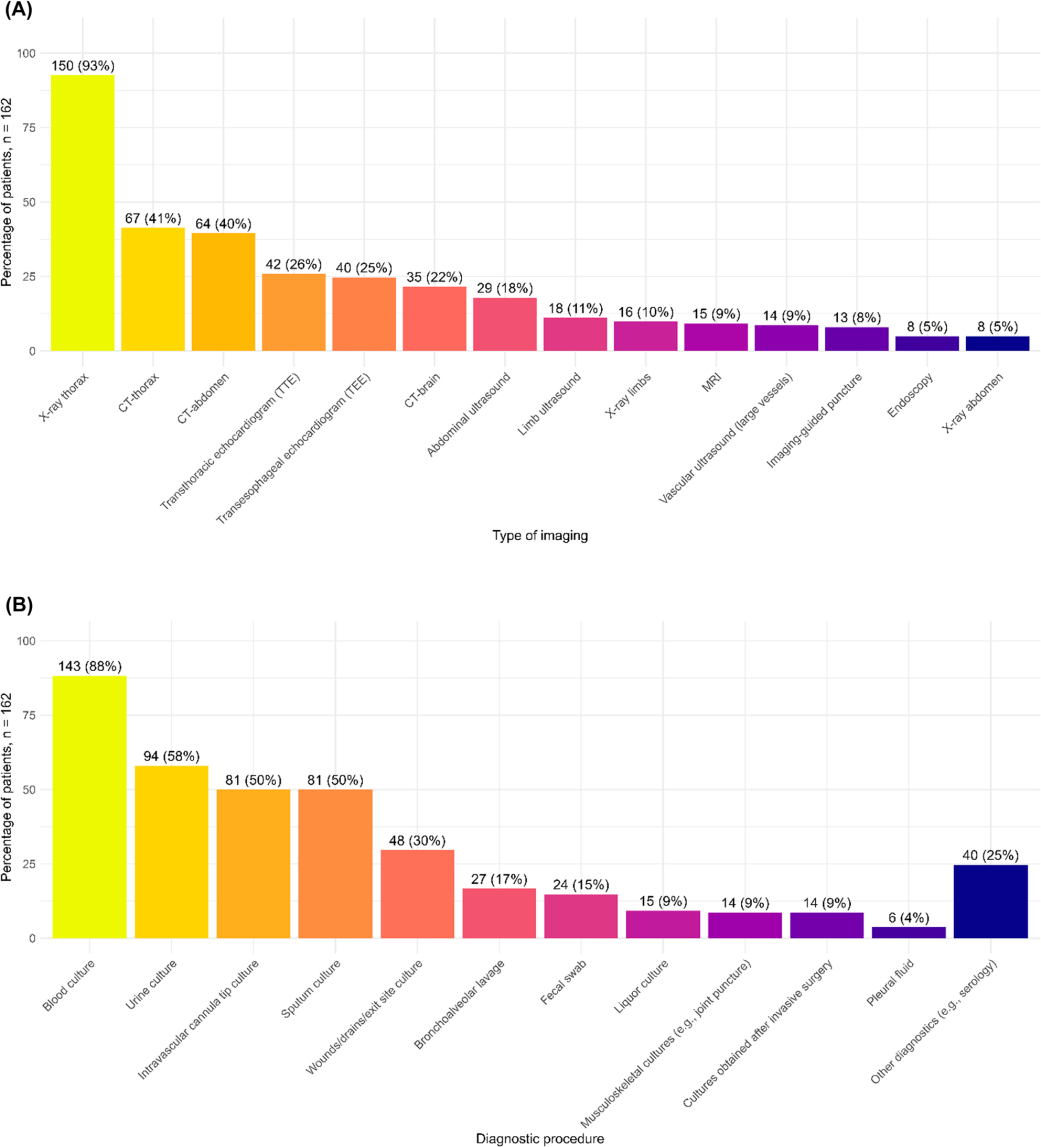
(**A**) Imaging in the week before (first) [^18^F]FDG PET/CT request. (**B**) Microbiological tests performed in the week before (first) [18F]FDG PET/CT request

During ICU admission, patients underwent a median of 7 (IQR 5–8) distinct diagnostic procedures prior to the [^18^F]FDG PET/CT scan, consisting of 3 (IQR 2–4) imaging procedures and 4 (IQR 3–5) microbiological tests (Fig. 2A and B). The diagnostic work-up before [^18^F]FDG PET/CT often included a conventional chest X-ray (150/162 indications; 93%) or a thoracic CT scan (67/162; 41%). Abdominal CT was obtained in 64 indications (40%), transthoracic echocardiography in 42 (26%), and transesophageal echocardiography in 40 (25%).

Blood cultures were obtained in the week prior to [^18^F]FDG PET/CT in 143/162 indications (88%), yielding positive results in 107/162 (66%). The most frequently isolated pathogens were *Staphylococcus aureus*, coagulase-negative staphylococci (e.g., *S. epidermidis*, not considered contaminants [26]), and *Enterococcus faecium*. Intravascular catheter-tip cultures and sputum cultures were performed in 81/162 patients (50%), and bronchoalveolar lavage (BAL) in 27/162 patients (17%).

### Diagnostic yield

In 109/162 indications (67%), [^18^F]FDG PET/CT yielded a conclusive diagnosis, of which 79 were newly identified (49%; Table 2). The most common new findings were musculoskeletal infections (e.g., septic arthritis, muscle abscess; 24%), respiratory tract infections (e.g., pneumonia, pulmonary abscess; 20%), and cardiac infections (e.g., endocarditis, pericarditis; 6%). A list of all new diagnoses is available in Supplementary Table 1. Therapy changes after [^18^F]FDG PET/CT were made in 70/162 of scan indications (43%; Table 2). The associations between diagnostic yield, ICU mortality, and length of stay are presented in Supplementary Table 2.

**Table 2:** Diagnostic yield of [^18^F]FDG PET/CT

No relevant findings	n = 162 ^1^
	53 (33)
**Confirmation of suspected diagnosis** ^**2**^	30 (19)
**Detection of new**,** previously unsuspected diagnosis** ^**2,3**^	79 (49)
* Musculoskeletal*	39 (24)
* Respiratory tract*	31 (19)
* Abdominal*	9 (6)
* Heart*	9 (6)
* Vascular system*	8 (5)
* Skin & soft tissues*	5 (3)
* Urinary tract*	4 (2)
* Central nervous system*	2 (1)
* Other*	5 (3)
**Therapeutic consequence** ^**3**^	70 (43)
* Start medication/prolong duration*	32 (20)
* Stop medication*	16 (10)
* Therapeutic intervention (e.g.*,* surgery)*	25 (15)
* Restrictions on care*	7 (4)

Presented as Median [IQR] or n (%)

^1^ Non-interpretable scans repeated due to poor quality (*n* = 7) were excluded

^2^ Only findings that provided an adequate explanation for the clinical indication of the scan (i.e., suspected infection or inflammation) were scored. Incidental or unrelated findings, including newly detected malignancies or chronic conditions, were not counted

^3^ Findings from one scan can contribute to multiple subcategories, totals therefore exceed 100%

In multivariable logistic regression analysis, diabetes mellitus (adjusted odds ratio [aOR] 0.35; 95% CI 0.15–0.80) and partial-body [^18^F]FDG PET/CT (versus total-body; aOR 0.48; 95% CI 0.24–0.99) were associated with lower odds of identifying a new diagnosis (Table 3). Use of Renal replacement therapy (RRT) was associated with the identification of more new diagnoses (aOR 2.32; 95% CI 0.93–5.75), although this association did not reach statistical significance at the 0.05 level. A longer interval between ICU admission and [^18^F]FDG PET/CT was independently associated with a therapy change (aOR 1.03 per day; 95% CI 1.01–1.05). No other variables were significantly associated with therapy changes.

**Table 3:** Crude (univariable) and adjusted (multivariable) regression analysis of diagnostic yield associated with [^18^F]FDG PET/CT

Variable	New diagnosis		Therapy change	
	Crude OR	Adjusted OR	Crude OR	Adjusted OR
Male sex (at birth)	1.42 (0.72–2.84)	1.47 (0.68–3.18)	1.33 (0.67–2.69)	1.39 (0.63–3.05)
Age (per 10 years)	1.01 (0.83–1.24)	1.08 (0.86–1.35)	0.96 (0.79–1.17)	1.03 (0.82–1.29)
BMI (per unit)	1.03 (0.97–1.09)	1.05 (0.98–1.12)	1.00 (0.94–1.06)	1.01 (0.94–1.08)
Diabetes mellitus	0.54 (0.26–1.08)	0.35 (0.15–0.80)*	1.07 (0.53–2.14)	1.18 (0.52–2.64)
Partial-body scan	0.66 (0.36–1.24)	0.48 (0.24–0.99)*	0.74 (0.39–1.38)	0.70 (0.34–1.43)
Fever > 38.3 °C ^1^	1.56 (0.83–2.97)	1.74 (0.82–3.70)	1.34 (0.71–2.54)	1.63 (0.76–3.50)
Use of RRT ^1^	1.65 (0.83–3.34)	2.32 (0.93–5.75)	2.58 (1.29–5.29)*	1.99 (0.81–4.90)
CRP (per 10 mg/L) ^2^	1.02 (0.99–1.05)	1.03 (0.99–1.06)	1.00 (0.97–1.03)	1.01 (0.98–1.04)
Leukocyte count (per 10^9/L) ^2^	0.99 (0.95–1.02)	0.98 (0.94–1.02)	0.99 (0.95–1.02)	0.99 (0.95–1.03)
SOFA score ^2^	1.03 (0.94–1.13)	0.99 (0.89–1.11)	1.05 (0.96–1.15)	1.07 (0.96–1.20)
Positive blood cultures ^1^	1.03 (0.54–1.98)	1.33 (0.63–2.81)	0.66 (0.34–1.26)	0.71 (0.34–1.51)
Microbiological work-up ^3^	1.04 (0.83–1.31)	1.04 (0.79–1.35)	1.14 (0.91–1.45)	1.18 (0.89–1.55)
Radiology work-up ^3^	1.01 (0.84–1.23)	1.03 (0.81–1.30)	0.86 (0.70–1.05)	0.92 (0.72–1.18)
Duration of antimicrobial therapy pre-scan (days)	1.00 (0.98–1.02)	0.99 (0.97–1.02)	1.02 (1.00–1.04)	1.00 (0.97–1.03)
Length of stay pre-scan (days)	1.00 (0.99–1.02)	1.00 (0.98–1.03)	1.03 (1.01–1.05)*	1.03 (1.00–1.05)*

Presented as Odds ratio (95% CI). Adjusted odds ratios were derived from multivariable logistic regression models including all variables listed in the table. BMI = Body mass index, RRT = Renal replacement therapy, CRP = C-reactive protein; SOFA = Sequential organ failure assessment

* Statistically significant at an alpha of 0.05

^1^ In week before scan request

^2^ On day of scan request

^3^ Number of unique procedures in the week before scan request

### Scan quality, myocardial suppression & optimal preparation

Optimal scan quality was observed in 72/169 scans (43%), and optimal myocardial suppression in 66/169 scans (39%). Interobserver agreement was moderate for both endpoints, with a linear weighted Cohen’s κ of 0.46 for scan quality and 0.50 for myocardial suppression.

In multivariable logistic regression, higher BMI was independently associated with increased odds of optimal scan quality (aOR 1.08; 95% CI 1.00 to 1.17; Table 4). Higher serum creatinine concentration (log-transformed) was associated with lower odds of optimal scan quality (aOR, 0.36; 95% CI, 0.20 to 0.68). Diabetes mellitus (aOR, 0.40; 95% CI, 0.16 to 1.00) and mechanical ventilation during the scan (aOR, 0.42; 95% CI, 0.17 to 1.01) were both associated with lower odds of optimal scan quality, although these associations did not reach statistical significance at the 0.05 level.

**Table 4:** Crude (univariable) and adjusted (multivariable) regression analysis of scan quality and myocardial suppression

Variable	Scan quality		Myocardial suppression	
	Crude OR	Adjusted OR	Crude OR	Adjusted OR
Male sex (at birth)	0.58 (0.30–1.15)	0.79 (0.35–1.80)	0.47 (0.24–0.93)*	0.45 (0.21–0.96)*
Age (per 10 years)	0.82 (0.67–1.00)	0.95 (0.73–1.23)	0.89 (0.73–1.09)	0.96 (0.76–1.20)
BMI (per unit)	1.03 (0.97–1.09)	1.08 (1.00–1.17)*	1.01 (0.96–1.07)	1.00 (0.94–1.07)
Diabetes mellitus	0.39 (0.18–0.79)*	0.40 (0.16–1.00)	0.91 (0.45–1.81)	1.10 (0.47–2.61)
Immunodeficiency	0.85 (0.33–2.06)	0.77 (0.24–2.46)	0.81 (0.31–1.99)	0.72 (0.23–2.21)
Any malignancy	1.01 (0.44–2.29)	1.24 (0.47–3.25)	0.57 (0.22–1.35)	0.54 (0.20–1.47)
Pre-scan glucose	0.89 (0.78–1.01)	0.97 (0.83–1.14)	0.99 (0.88–1.11)	1.03 (0.89–1.20)
Pre-scan creatinine ^1^	0.46 (0.29–0.71)*	0.36 (0.20–0.68)*	0.99 (0.96–1.01)	0.99 (0.95–1.02)
Use of sedation during scan	1.10 (0.58–2.08)	0.42 (0.17–1.01)	0.89 (0.46–1.70)	1.55 (0.55–4.36)
Use of mechanical ventilation during scan	0.73 (0.39–1.38)	1.34 (0.56–3.18)	0.71 (0.38–1.35)	1.29 (0.51–3.27)
Fasting 12–24 h ^2^	0.87 (0.35–2.11)	1.37 (0.48–3.92)	1.01 (0.41–2.52)	1.03 (0.45–2.36)
Fasting > 24 h ^2^	0.54 (0.24–1.21)	0.82 (0.31–2.13)	0.97 (0.43–2.23)	0.60 (0.26–1.40)
SOFA score at time of scan	0.98 (0.90–1.08)	1.02 (0.92–1.14)	0.96 (0.88–1.05)	0.96 (0.87–1.07)
Corticosteroid use in week before scan	0.66 (0.36–1.22)	0.58 (0.27–1.27)	0.78 (0.42–1.45)	0.78 (0.38–1.63)
Bolus of insulin on day of scan	0.32 (0.05–1.32)	0.38 (0.06–2.50)	0.65 (0.14–2.44)	0.41 (0.08–2.07)
Pre-scan bolus administration of unfractionated heparin	0.90 (0.46–1.77)	1.07 (0.47–2.43)	0.52 (0.26–1.07)	0.43 (0.19–0.98)*

Presented as Odds ratio (95% CI). Adjusted odds ratios were derived from multivariable logistic regression models including all variables listed in the table. BMI = Body mass index, RRT = Renal replacement therapy, CRP = C-reactive protein; SOFA = Sequential organ failure assessment

* Statistically significant at an alpha of 0.05

^1^ Log-transformed creatinine in the scan-quality analysis to achieve linearity, OR per 10 µmol/L increase of creatinine in the myocardial suppression analysis

^2^ Also includes carbohydrate free diet. Reference category: <12 h

Male sex (aOR, 0.45; 95% CI, 0.21 to 0.96) and a pre-scan bolus administration of unfractionated heparin (aOR, 0.43; 95% CI, 0.19 to 0.98) were independently associated with lower odds of achieving optimal myocardial suppression. Notably, pre-scan glucose was not significantly associated with either scan quality or myocardial suppression.

## Discussion

In this retrospective multicenter cohort study, we found that [^18^F]FDG PET/CT frequently identified previously unrecognized conditions and often prompted therapy changes in critically ill patients suspected of IUO, even after conventional work-up. Total-body imaging, the absence of diabetes, and a longer ICU stay were independent determinants of greater diagnostic yield. Higher BMI and lower serum creatinine were associated with improved scan quality, and male sex and pre-scan heparin bolus were associated with suboptimal myocardial suppression.

Previous studies, each with small sample sizes (17–47 patients), and all but one single-center [4, 10–13], reported the identification of an infectious focus in 70–91% of indications, compared to 67% in this study. In studies reporting on new findings, 28–72% of diagnoses were previously unrecognized, compared to 49% in this study. Differences in reported detection rates of new diagnoses may partly reflect variation in patient populations and case definitions. Some earlier studies focused exclusively on bloodstream infections [13] or sepsis of unknown origin [10, 12], while others included patients with persistent critical illness later during ICU stay [4]. Reported therapy changes ranged from 15% to 71%, consistent with the 44% observed in our study. Only one study (*n* = 30) reported predictors of new diagnoses in critically ill patients [13]. In line with our findings, Pijl et al. reported no significant associations between new diagnoses and systemic inflammatory markers, ICU length of stay, or prior antibiotic therapy. However, diabetes and total versus partial-body imaging were not evaluated in that study.

Respiratory tract infections accounted for a substantial proportion of newly identified foci, despite 93% of patients having undergone chest radiography and 41% having undergone thoracic CT prior to PET/CT. One possible explanation is the limited sensitivity of chest radiography for pulmonary infection or inflammation in critically ill patients [27], whereas [^18^F]FDG PET/CT may improve detection through combined metabolic and anatomical imaging, as suggested in previous studies [28, 29]. Timing may also play a role, as pulmonary abnormalities could have developed between prior thoracic imaging and PET/CT. Finally, the true presence of pneumonia could not always be confirmed retrospectively, since bronchoalveolar lavage—when performed—often occurred under ongoing antibiotic therapy.

In this study, the observed associations between patient- and scan characteristics and diagnostic yield are clinically plausible. The absence of diabetes mellitus likely reflects more favorable glucose metabolism, thereby reducing [^18^F]FDG background uptake [30], while total-body imaging increases the probability of capturing peripheral sites of infection or inflammation. This is consistent with the observation that the musculoskeletal system was the most common site of new diagnoses in our cohort. The association between pre-scan length of stay and therapy changes after [^18^F]FDG PET/CT may reflect a greater willingness to de-escalate treatment later in the ICU course, when no (new) diagnoses are observed, though this cannot formally be concluded from our data. In our cohort, longer ICU stay before imaging was more frequently associated with cessation of antimicrobial therapy or restrictions on care, rather than treatment initiation or other therapeutic interventions (data not shown). This finding, therefore, likely reflects clinical decision-making later during ICU admission, and does not necessarily suggest that PET/CT should be positioned later in the diagnostic work-up.

Several patient- and procedural characteristics were associated with scan quality. Although BMI was not associated with scan quality in univariate analysis, a positive association emerged after adjustment for diabetes mellitus —a finding that contrasts with previous literature, where higher BMI is typically associated with increased noise and reduced quality [31]. This may reflect negative confounding, as diabetes—more prevalent among patients with higher BMI—showed a trend toward poorer scan quality. Elevated serum creatinine was significantly associated with poorer scan quality, consistent with prior reports linking renal dysfunction to altered tracer clearance and increased background activity [32, 33]. Myocardial [^18^F]FDG uptake was significantly higher in men than in women, reflecting known differences in myocardial glucose extraction fraction and utilization, and in line with previous studies [34, 35]. The most unexpected finding in our study was that pre-scan administration of unfractionated heparin was associated with *lower* odds of achieving adequate myocardial suppression, contrasting with current ASNC/SNMMI imaging guidelines, which recommend unfractionated heparin to suppress physiological myocardial [^18^F]FDG uptake [36]. While one study found that heparin pre-loading, when added to a low-carbohydrate diet, improved suppression of cardiac glucose metabolism in patients with suspected infection [37], multiple studies in patients with suspected cardiac sarcoidosis found no benefit of heparin in reducing myocardial [^18^F]FDG uptake [38–40]. The lack of efficacy observed in our critically ill population may be influenced by the high proportion of patients (82%) treated with prophylactic or therapeutic low-molecular-weight heparin (LMWH), which induces a more modest increase in free fatty acids compared to unfractionated heparin [41]. Moreover, the distribution of heparin bolus administration across participating centers was not balanced, with two centers accounting for 88% of all patients receiving pre-scan unfractionated heparin; thus, confounding by unmeasured center-level factors cannot be excluded. The distribution of study determinants across hospitals is available in Supplementary Table 3. Other possible explanations include alternative myocardial substrate preference, altered lipolytic response, and changes in heparin pharmacokinetics under critical illness, although our study was not designed to investigate these hypotheses [42–44]. Taken together, our findings suggest that routine administration of unfractionated heparin to improve myocardial suppression may not be beneficial—and could even be counterproductive—in the critically ill. However, this finding warrants prospective validation.

Pre-scan blood glucose concentration was not a significant predictor of scan quality or myocardial suppression in this study. This may be attributable to the limited variability in glucose levels, which ranged from 3.4 to 13.3 mmol/L in all but one case. The single outlier (pre-scan glucose level 21.4 mmol/L) was rated as poor quality by both observers. Point estimates for pre-scan insulin bolus administration were consistently in the direction of lower scan quality and myocardial suppression; however, confidence intervals were wide, reflecting limited exposure (*n* = 10), consistent with current guidelines discouraging pre-scan insulin administration [45]. Nearly all scans (98%) were preceded by a fasting period of at least 5 h. Although longer fasting duration was not associated with improved scan quality or myocardial suppression in this cohort, previous studies have reported improved image quality and myocardial suppression with prolonged fasting [38, 40]. However, the optimal duration of fasting remains unknown even though a large range of fasting durations (4–18 h) has been assessed [21].

Our study is the first large, multicenter study to evaluate determinants of diagnostic yield, image quality, and myocardial suppression of [^18^F]FDG PET imaging in the ICU. By including patients from both academic and non-academic teaching hospitals, the study captures heterogeneity in ICU populations, imaging protocols, and clinical practice (including differences in patient preparation). This improves external validity and enables the assessment of factors influencing scan quality across different institutional settings. Furthermore, the availability of complete clinical data on both determinants and outcomes strengthens the study’s internal validity. Limitations include the retrospective observational design, precluding causal inference and leaving potential residual confounding. The relative homogeneity in key preparation parameters (e.g., nearly all patients fasted ≥ 5 h and had pre-scan glucose < 13.3 mmol/L) may have attenuated potential associations. Second, although this is the largest study to date, the sample size remains insufficient for reliable individual-level prediction. The multivariable models are likely overfitted and not suitable to guide individual clinical decisions. However, our primary aim was to quantify diagnostic yield and identify factors associated with diagnostic yield and scan quality. Lastly, agreement between assessors was moderate for both scan quality and myocardial suppression. This is in line with prior studies reporting on inter-observer agreement of [^18^F]FDG PET/CT quality, underscoring the challenges of subjective scoring in the absence of validated, standardized assessment tools [46, 47]. Moreover, due to the low number of scans rated as “poor,” it was not feasible to model scan quality as an ordinal outcome, which was therefore dichotomized for analysis.

Future research should focus on optimizing patient selection for [^18^F]FDG PET/CT and defining its optimal timing within the diagnostic work-up of critically ill patients suspected of IUO. Standardized protocols for patient preparation in the ICU—including fasting duration, glucose management, and myocardial suppression strategies—should be prospectively evaluated to improve image quality and interpretability. Finally, future studies should also assess the cost-effectiveness of [^18^F]FDG PET/CT in this population.

## Conclusion

[^18^F]FDG PET/CT identified a new diagnosis in 49% of cases and led to therapy changes in 43% of critically ill patients suspected of IUO. Most new diagnoses were identified in the musculoskeletal system and the respiratory tract. Scan quality and myocardial [^18^F]FDG uptake were influenced by patient characteristics and preparation. Optimizing these factors may further enhance the clinical utility of [^18^F]FDG PET/CT in the ICU.

## Supplementary Information

Below is the link to the electronic supplementary material.

Supplementary Material 1.

## Data Availability

Study data and code are available upon reasonable request sent to the corresponding author.
