# Supplementary material for: Determinants of diagnostic yield and scan quality of [18F]FDG PET/CT in critically ill patients suspected of infection or inflammation of unknown origin
Source: Crit Care. 2026 Apr 10;30:256. doi: 10.1186/s13054-026-06005-w (PMC13173825; doi:10.1186/s13054-026-06005-w)
Supplement: Supplementary file 1 — Supplementary Material 1. [file 13054_2026_6005_MOESM1_ESM.docx]

**Supplementary table 1.** **New Acute Diagnoses Identified by [^18^F]FDG PET/CT**

| **Localization** | **Diagnosis** | **n (162)** |
| --- | --- | --- |
| Musculoskeletal (n = 39) | (Septic) Arthritis  Muscle abscess  Bursitis  Myositis  Spondylodiscitis  Unclear (aspecific muscular activity)  Joint prosthesis infection  Synovitis  Vertebral abscess  Polymyalgia rheumatica | 15  14  8  6  5  3  3  2  1  1 |
|  |  |  |
| Respiratory tract (n = 32) | Pneumonia  Aspecific nodus (likely infectious)  Pulmonary aspergilloma  Pleuritis  Pleural empyema  Radiation pneumonitis  Interstitial lung disease | 24  3  1  1  1  1  1 |
|  |  |  |
| Heart (n = 9) | Endocarditis  Bentall prosthesis infection  Pericarditis  Paravalvular abscess | 5  2  2  1 |
| Abdomen (n = 9) | Abdominal abscess  Pancreatitis  Colitis  Gut perforation/anastomotic leakage  Gastritis  Diverticulitis | 3  3  1  1  1  1 |
|  |  |  |
| Vascular system (n = 8) | Septic embolism  Thrombophlebitis  Infected thrombus  Aortitis | 4  2  1  1 |
| Skin/Soft tissues (n = 5) | Aspecific skin defect (likely infectious)  Infected decubitus  Infected subcutaneous hematoma  Erysipelas/cellulitis | 2  1  1  1 |
| Urinary/reproductive tract (n = 4) | Prostatitis  Infected renal cysts  Pyelonephritis | 2  1  1 |
| Central nervous system (n = 2) | Encephalitis  Epidural abscess | 1  1 |
| Drains/Leads/Devices (n = 2) | Pacemaker + lead infection  Left ventricular assist device (LVAD) infection | 1  1 |
| Other (n = 3) | Mediastinitis  Sinusitis | 2  1 |

**Supplementary table 2. Association between diagnostic yield and patient-centered outcomes**

| **Diagnosis** | **In-ICU mortality** | **ICU length of stay** |
| --- | --- | --- |
| No relevant findings (n = 53) | 14 (26) | 22 [6–53] |
| Known diagnosis (n = 30) | 7 (23) | 28.5 [15.5–56.5] |
| New diagnosis (n = 79) | 26 (33) | 29 [14–49.5] |
| **Therapy change** |  |  |
| No therapy change (n = 92) | 23 (25) | 22 [9–39] |
| Cessation of medication (n = 16) | 4 (25) | 45 [33–76] |
| Start or prolongation of medication (n = 32) | 9 (28) | 37 [17–52] |
| Intervention e.g., surgery/drainage (n = 25) | 10 (40) | 42 [13–75] |
| Restrictions on care (n = 7) | 7 (100) | 25 [7–42] |
| Presented as Median [IQR] or n (%). |  |  |

**Supplementary table 3. Distribution of study determinants across participating sites**

|  | **Participating sites** | | | |
| --- | --- | --- | --- | --- |
| **Variable** | **UMCU  (n = 56)** | **Amphia Hospital  (n = 43)** | **St. Antonius Hospital (n = 28)** | **UMCG  (n = 42)** |
| Male sex (at birth) | 41 (73) | 32 (74) | 25 (89) | 23 (55) |
| Age | 60 [48–69] | 68 [61–72] | 68 [63–75] | 59 [46–65] |
| BMI | 27.1 [24.7–29.2] | 27.1 [24.7–30.2] | 26.9 [22.5–30.3] | 28.6 [25.6–31.5] |
| Diabetes mellitus | 17 (30) | 16 (37) | 5 (18) | 10 (24) |
| Immunodeficiency | 13 (23) | 2 (5) | 2 (7) | 6 (14) |
| Any malignancy | 13 (23) | 7 (16) | 7 (25) | 1 (2) |
| Partial-body scan | 9 (16) | 14 (33) | 24 (86) | 29 (69) |
| Fever >38.3°C ^1^ | 23 (41) | 22 (51) | 9 (32) | 13 (31) |
| Use of RRT ^1^ | 15 (27) | 12 (28) | 8 (29) | 20 (48) |
| CRP (per 10 mg/L) ^2^ | 118 [59.5–179] | 140 [77.5–194] | 176.5 [54–243] | 193 [107–297] |
| Leukocyte count ^2^ | 12.1 [7.9–17.3] | 12.5 [7.8–17] | 14.2 [10.3–17.3] | 13.2 [10.1–18.4] |
| SOFA score ^2^ | 6 [5–8] | 6 [4–8] | 7 [5–9] | 8.5 [4–11] |
| Positive blood cultures ^1^ | 38 (68) | 25 (58) | 23 (82) | 27 (64) |
| Microbiological work-up ^3^ | 4 [3–5] | 4 [3–5] | 3.5 [3–5] | 4 [3-4] |
| Radiology work-up ^3^ | 3 [2-4] | 3 [2-4] | 2.5 [2-4] | 3 [2-5] |
| Duration of antimicrobial therapy pre-scan (days) | 10.5 [7–26.5] | 6 [3-7] | 7.5 [3–11.5] | 7 [5–14] |
| Length of stay pre-scan (days) | 14 [8–26] | 7 [4–16.5] | 14 [5–34.5] | 8 [4–21] |
| Pre-scan glucose | 6.4 [5.3–8.3] | 7.1 [5.9–8.9] | 5.6 [4.8–6.9] | 5.8 [5.1–7.8] |
| Pre-scan creatinine | 83 [59–122] | 111 [70–198] | 122 [65.5–200.5] | 114 [56–206.5] |
| Use of sedation during scan | 18 (32) | 17 (40) | 8 (29) | 16 (38) |
| Use of mechanical ventilation during scan | 42 (75) | 25 (58) | 16 (57) | 26 (60) |
| Fasting <12 hours | 4 (7) | 9 (21) | 8 (29) | 12 (29) |
| Fasting 12-24 hours | 16 (29) | 15 (35) | 6 (21) | 11 (26) |
| Fasting >24 hours | 36 (64) | 19 (44) | 14 (50) | 19 (45) |
| Corticosteroid use in week before scan | 21 (38) | 31 (72) | 10 (36) | 16 (38) |
| Bolus of insulin on day of scan | 9 (16) | 1 (2) | 0 (0) | 0 (0) |
| Pre-scan bolus administration of unfractionated heparin | 12 (21) | 31 (72) | 6 (21) | 1 (2) |

Presented as Median [IQR] or n (%).

^1^ In week before scan request

^2^ On day of scan request

^3^ Number of unique procedures in the week before scan request
